# Supplementary material for: Investigation of the Influence of PLA Molecular and Supramolecular Structure on the Kinetics of Thermal-Supported Hydrolytic Degradation of Wet Spinning Fibres
Source: Materials (Basel). 2020 May 2;13(9):2111. doi: 10.3390/ma13092111 (PMC7254213; doi:10.3390/ma13092111)
Supplement: Supplementary file 1 [file materials-13-02111-s001.pdf]

materials

MDPI

Supplementary Files

Investigation of the Influence of PLA Molecular and Supramolecular Structure on the Kinetics of Thermal-Supported Hydrolytic Degradation of Wet Spinning Fibres

Małgorzata Giełdowska, Michał Puchalski, Grzegorz Szparaga and Izabella Krucińska

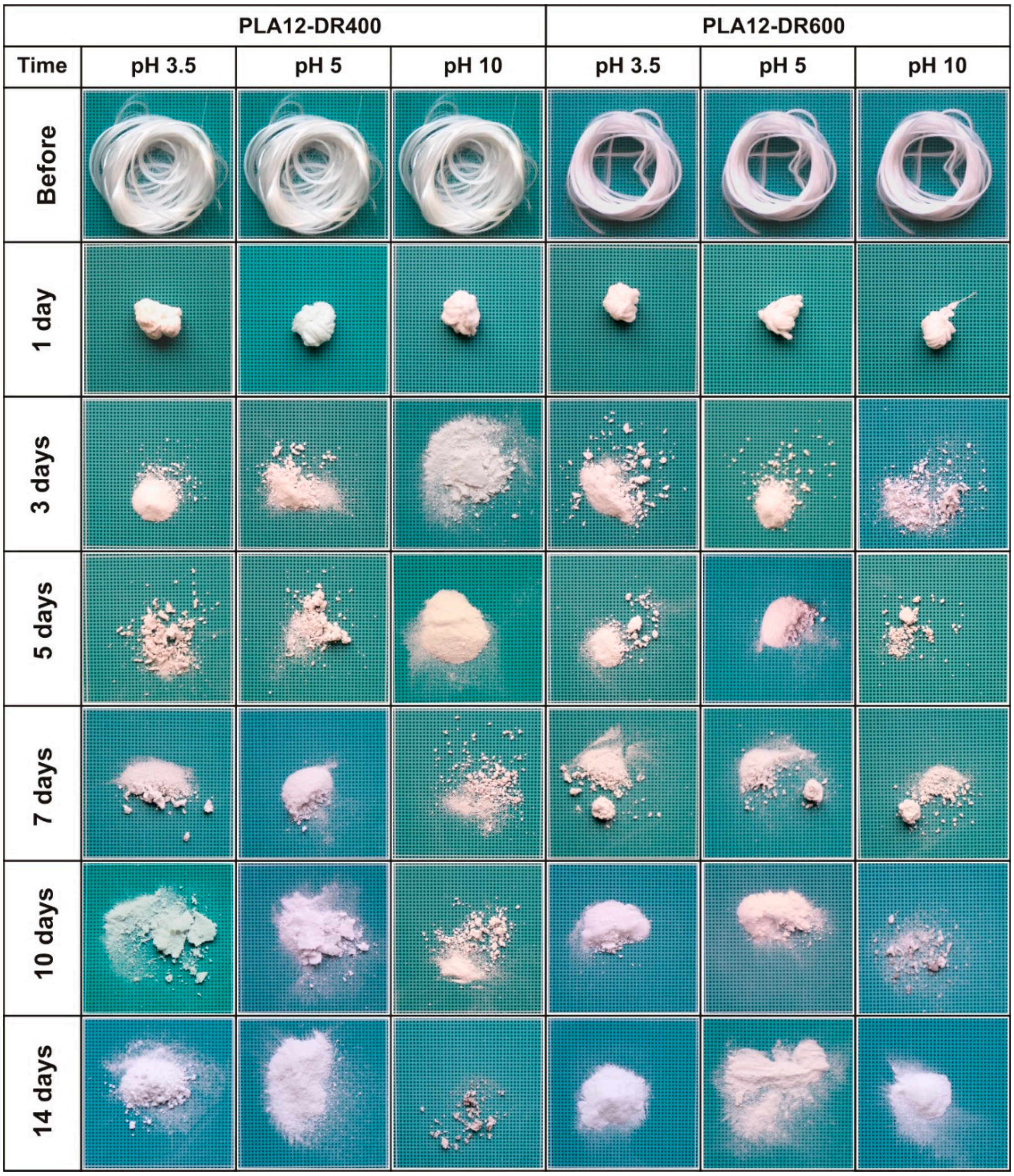

Figure S1. Photographic documentation of thermal-supported hydrolytic degradation of fibres obtained from PLA containing 12% D-lactide isomer.

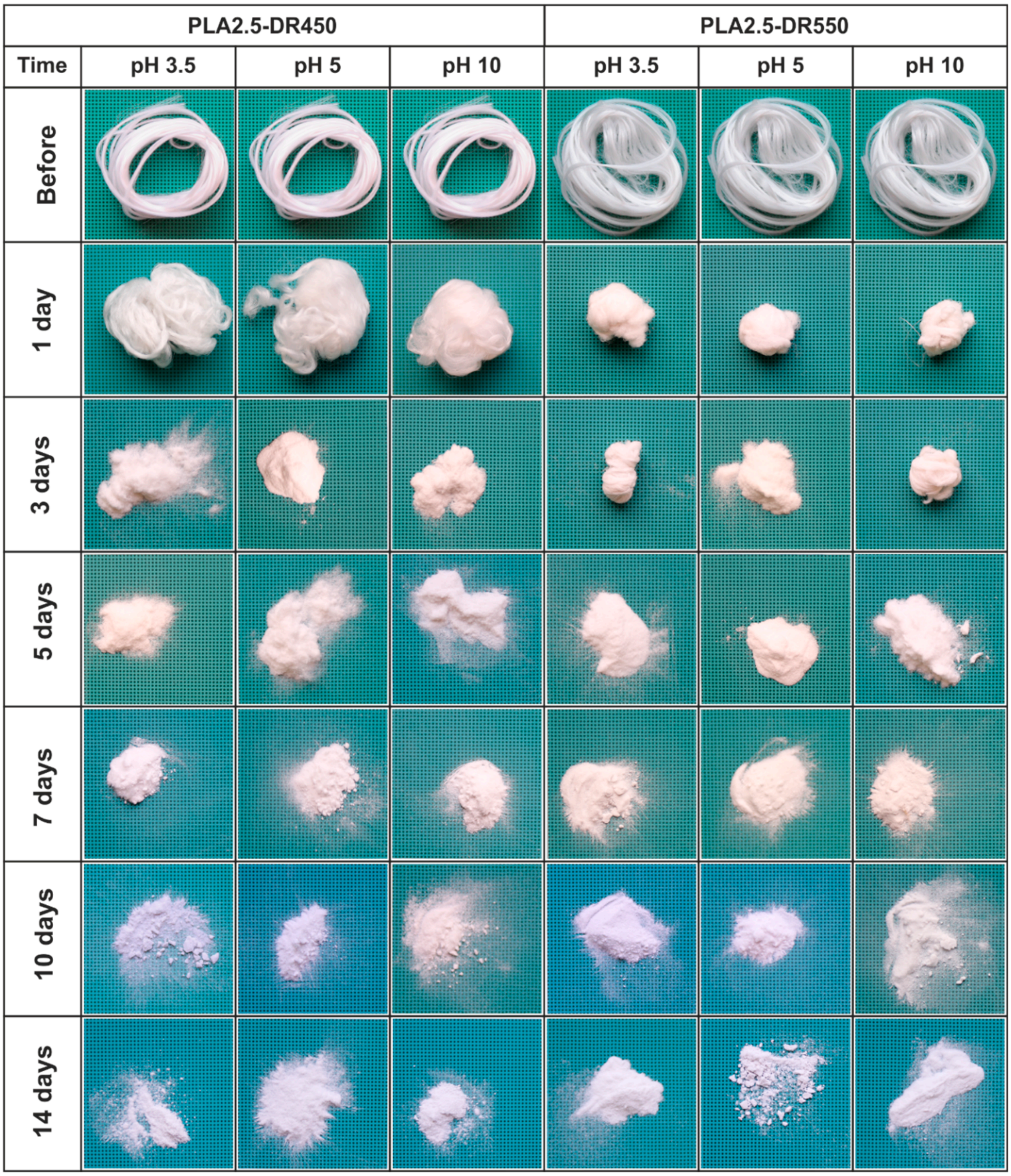

Figure S2. Photographic documentation of thermal-supported hydrolytic degradation of fibres obtained from PLA containing 2.5% D-lactide isomer.

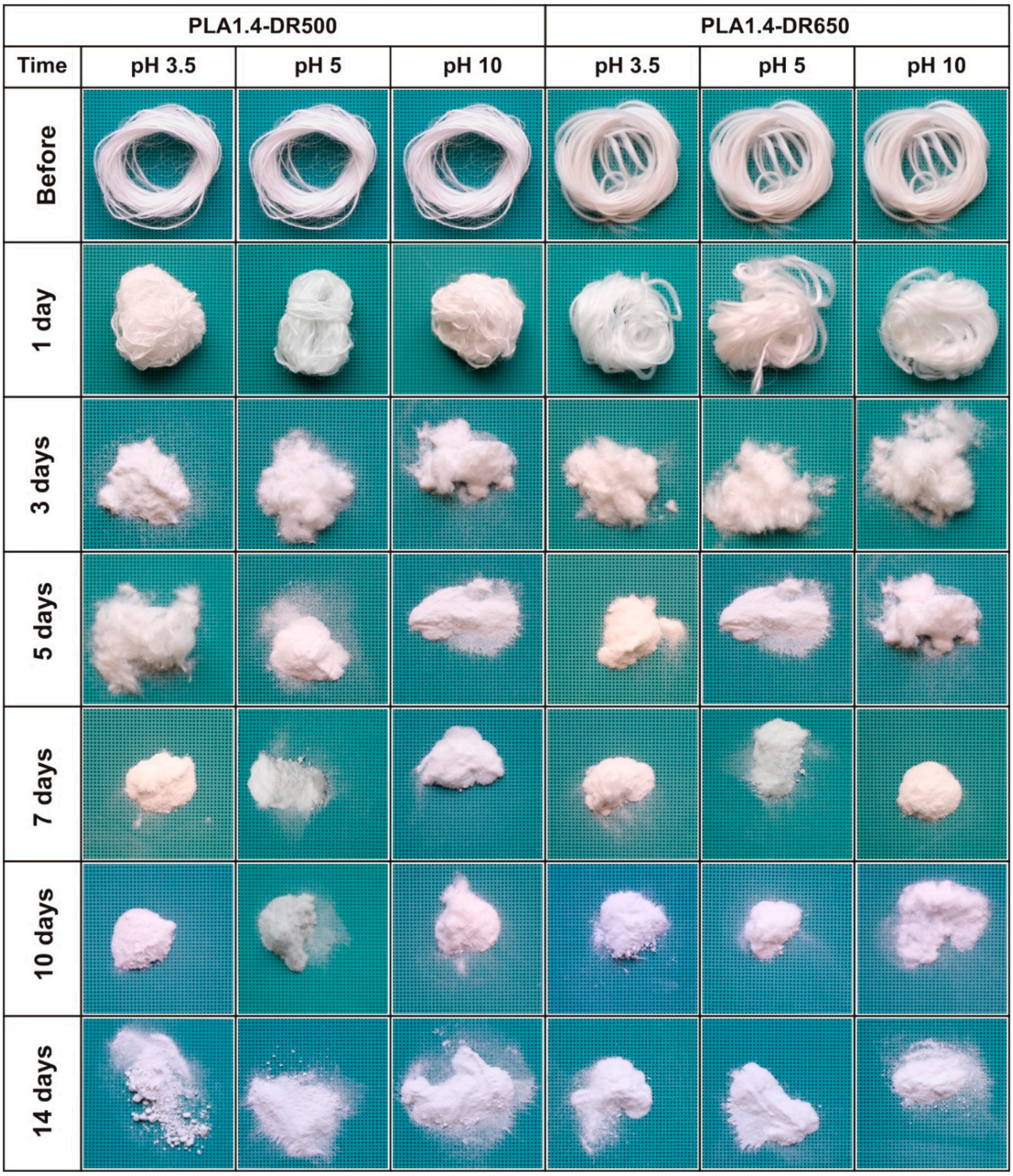

Figure S3. Photographic documentation of thermal-supported hydrolytic degradation of fibres obtained from PLA containing 1.4% D-lactide isomer.
